# Supplementary material for: A Single-cell Atlas of Developing Mouse Palates Reveals Cellular and Molecular Transitions in Periderm Cell Fate
Source: Genomics Proteomics Bioinformatics. 2025 Mar 4;23(1):qzaf013. doi: 10.1093/gpbjnl/qzaf013 (PMC12240470; doi:10.1093/gpbjnl/qzaf013)
Supplement: qzaf013_Supplementary_Data [file qzaf013_supplementary_data.zip › supplementary material captions.docx]

**Supplementary material**

**Figure S1 scRNA-seq of 41,419 cells from the developing palate in mice across four critical developmental stages**

The stages include E10.5 (initial stage), E13.5 (vertical growth), E15.0 (fusion initiation), and E16.5 (completion). Palatal tissue at each stage was microscopically isolated, with the maxillary prominence and surrounding tissue isolated for E10.5. Subsequently, the isolated tissues were digested and subjected to scRNA-seq using the 10X Chromium system. The dashed circles denote the regions of isolated tissue in representative images of sampled embryos. Five embryos were microscopically isolated for analysis at each time point. EDTA, ethylenediaminetetraacetic acid; GEMs, gel beads in emulsion.

**Figure S2 Quality control and sequencing information**

**A.** Percentage of viable cells in samples at each time point following tissue digestion. **B.** Number of detected genes per cell at each time point. **C.** Number of detected UMI counts per cell at each time point. **D.** Sequencing details for scRNA-seq of samples at four time points. UMI, unique molecular identifier.

**Figure S3 Heatmap highlighting relative expression levels of key marker genes across cell types and clusters**

The color scale represents the expression level. The upper bars indicate the major cell types, while the lower bars correspond to the cell clusters listed in Figure 1.

**Figure S4 UMAP plots with overlaid expression of cluster-specific genes in mesenchymal cells (A), epithelial cells (B), and other cells (C)**

The color scale indicates the expression levels of the marker genes.

**Figure S5 Immunofluorescence validation of mesenchymal, epithelial, and periderm cell populations identified by scRNA-seq**

**A.–C.** Coronal sections of E10.5, E13.5, E15.0, and E16.5 mouse embryos were analyzed using immunofluorescence assays for COL3A1 (marker gene for mesenchymal cell population) (A), TRP63 (marker gene for epithelial cell population) (B), and KRT6A (marker gene for periderm cell population) (C). The results confirmed the presence of these cell populations. Notably, at E10.5, no KRT6A-positive cells were observed. However, from E13.5 to E16.5, there was a marked increase in KRT6A expression in periderm cells, consistent with our scRNA-seq analysis (refer to Figure S10). Dashed boxed areas are shown at higher magnification in the middle and bottom panels of A, B, and C. White dashed lines indicate the boundary between the epithelium and mesenchyme, and yellow arrowheads indicate KRT6A-positive cells. Nuclei are stained with DAPI. Scale bars, 50 μm. MxP, maxillary prominence.

**Figure S6 Distribution of cell clusters during palatogenesis in mice**

**A.** Fraction of cell clusters at each time point, demonstrating an incremental cell-cluster complexity as the palate develops. **B.** Bar plot illustrating the percentage of cells at each stage in each cell cluster, split by samples collected at different time points.

**Figure S7 Pseudotime reconstruction of mesenchymal development, illustrated separately by samples collected at different time points, which refer to Figure 2B**

**Figure S8 Pseudotime reconstruction of epithelial development, illustrated separately by samples collected at different time points, which refer to Figure 3B**

**Figure S9 Sub-clustering and annotation of C6 (*Krt6+* cells)**

**A.** Heatmap highlighting key marker genes utilized for inferring periderm population in C6. The color scale represents the expression level. **B.** Summary of subpopulations of C6.

**Figure S10 Characterization of marker genes expression in subclusters of periderm cells**

**A.** Dot plot illustrating the relative expression of selected marker genes in each subcluster. The dot size represents the percentage of cells within a cell cluster where the relevant markers were detected. The color indicates the average expression level. **B.** UMAP plots with overlaid expression of subcluster-specific genes, related to Figure 4D and E. The color scale indicates the expression level of the marker genes. **C.** Kinetics plots displaying the relative expression of marker genes for periderm subpopulations across developmental pseudotime.

**Figure S11 Immunofluorescence validation of keratinized periderm cells I (C6.5.0) identified by scRNA-seq**

Coronal sections of E13.5, E15.0, and E16.5 mouse embryos were analyzed using immunofluorescence assays for KRT10. At E13.5, no KRT10-positive cells were detected. However, at E15.0 and E16.5, numerous KRT10-positive cells were observed on the epithelial surface, with a slightly higher expression at E15.0 compared to E16.5. This expression pattern of KRT10 confirmed the identification of C6.5.0 as keratinized periderm cells I (refer to Figure 4 and Figure S10). Dashed boxed areas are shown at higher magnification in the middle and bottom panels. White dashed lines indicate the boundary between the epithelium and mesenchyme, and yellow arrowheads indicate KRT10-positive cells. Nuclei are stained with DAPI. Scale bars, 50 μm.

**Figure S12 Immunofluorescence validation of keratinized periderm cells II (C6.5.1) identified by scRNA-seq**

Coronal sections of E13.5, E15.0, and E16.5 mouse embryos were analyzed using immunofluorescence assays for KLF4. At E13.5 and E15.0, only a few KLF4-positive cells were detected. However, at E16.5, there was a significant increase in KRT10-positive cells on the epithelial surface. The expression pattern of KLF4 was consistent with scRNA-seq analysis, confirming the identification of C6.5.1 as keratinized periderm cells II (refer to Figure 4 and Figure S10). Dashed boxed areas are shown at higher magnification in the middle and bottom panels. White dashed lines indicate the boundary between the epithelium and mesenchyme, and yellow arrowheads indicate KLF4-positive cells. Nuclei are stained with DAPI. Scale bars, 50 μm.

**Figure S13 Immunofluorescence validation of medial edge periderm cells (C6.5.2) identified by scRNA-seq**

Coronal sections of E13.5, E15.0, and E16.5 mouse embryos were analyzed using immunofluorescence assays for IGFBP3. IGFBP3 was primarily expressed in the MES region at E15.0, while it was rarely detected in the epithelium region at both E13.5 and E16.5. These findings confirm the identification of C6.5.2 as medial edge periderm cells (refer to Figure 4 and Figure S10). Dashed boxed areas are shown at higher magnification in the middle and bottom panels. White dashed lines indicate the boundary between the epithelium and mesenchyme, and yellow arrowheads indicate IGFBP3-positive cells. Nuclei are stained with DAPI. Scale bars, 50 μm.

**Figure S14 Immunofluorescence validation of primitive periderm cells (C6.5.3) identified by scRNA-seq**

**A.** and **B.** Coronal sections of E13.5, E15.0, and E16.5 mouse embryos were analyzed using immunofluorescence assays for ARHGAP29 (A) and CLDN3 (B). At E13.5, both ARHGAP29-positive and CLDN3-positive cells were prominently observed on the epithelial surface of the palatal shelves. In contrast, at E15.0 and E16.5, few ARHGAP29-positive and CLDN3-positive cells were detected on the epithelial surface. The expression patterns of ARHGAP29 and CLDN3 confirmed the identification of C6.5.3 cells as primitive periderm cells (refer to Figure 4 and Figure S10). Dashed boxed areas are shown at higher magnification in the middle and bottom panels of A and B. White dashed lines indicate the boundary between the epithelium and mesenchyme, and yellow arrowheads indicate ARHGAP29-positive and CLDN3-positive cells. Nuclei are stained with DAPI. Scale bars, 50 μm.

**Figure S15 GRN displaying key regulators during periderm cell differentiation**

**A.** GRN for the trajectory of fusing. **B.** GRN for the trajectory of keratinizing. The GRN consists of 14 & 28 TFs expressed dynamically across the pseudotime of the trajectory of fusing and the pseudotime of the trajectory of keratinizing, respectively. Arrows in orange indicate activation, while arrows in blue represent repression. Node size indicates the number of predicted connections. The stages (Stage 1–3) correspond to Figure 5A and B.

**Figure S16 PITX2 knockdown impairs adhesion of mouse palatal shelves *ex vivo***

**A.** The silencing sequence of *Pitx2* KD-Ad and its targeting sites are shown. Note that the two nucleotides marked in red in human do not form complementary pairs with *Pitx2* KD-Ad silencing sequence. **B.** The results of RT-qPCR revealed a significant reduction in *Pitx2* mRNA in both mouse (C2C12) and human (GMSM-K) cell lines after *Pitx2* KD-Ad treatment. Each assay was performed in biological triplicate. **, *P* < 0.01; ***, *P* < 0.001. **C.** and **D.** The state of palatal adhesion was evaluated using HE staining after 72 h of palatal organ culture. Samples were treated with NC-Ad **(C)** or *Pitx2* KD-Ad (D). **E.** and **F.** IHC assays were conducted to detect PITX2 expression levels in palatal shelves after 72 h of culture. Samples were treated with NC-Ad (E) or *Pitx2* KD-Ad (F). In the *Pitx2* KD-Ad group, PITX2 expression in the periderm was reduced compared to that in the NC-Ad group. Pound signs indicate non-adhesion of palatal shelves. Scale bars, 50 μm. *Pitx2* KD-Ad, *Pitx2* knockdown adenovirus; mRNA, messenger RNA; RT-qPCR, quantitative real-time polymerase chain reaction; NC-Ad, the negative control adenovirus; HE, hematoxylin–eosin; IHC, immunohistochemistry.

**Figure S17 Immunofluorescence validation of EMT, apoptosis, and migration contributing to the degeneration of periderm cells in the MES**

**A.–C.** Coronal sections of E15.0 mouse embryos were analyzed using immunofluorescence assays for STMN1 (A), ANXA6 (B), and TAGLN (C). Our analysis revealed that STMN1 (an EMT marker) and ANXA6 (an apoptosis marker) were expressed within the MES region. TAGLN, a marker for cell migration, was expressed in the region abutting the oral side. The expression patterns of these markers were consistent with the results obtained from the RNAscope ISH assay for *Vim*, *Igfbp3*, and *Csrp1* (refer to Figure 7), confirming that EMT, apoptosis, and migration collectively contribute to the degeneration of periderm cells in the MES. White dashed lines indicate the MES region and arrows point to the STMN1-positive, ANXA6-positive, and TAGLN-positive cells. Nuclei are stained with DAPI. Scale bars, 20 μm.

**Figure S18 RNAscope ISH demonstrating the expression of *Pitx2* in the MES as well as widely in the oral epithelium**

*Pitx2* was strongly expressed at the junction site between maxillary and mandibular tissue, where the two layers of epithelial cells are merging into a single layer (white arrow in B). Note that the expression of *Pitx2* does not lead to widespread pathological adhesion in the oral region. Dashed boxed areas are shown at higher magnification in B. Scale bars, 50 μm. Max, maxillary tissue; Man, mandibular tissue.

**Table S1 Summary of cell clusters during mouse palatogenesis**

**Table S2 Differentially expressed genes for 5 developmental stages of mesenchymal cells**

**Table S3 Differentially expressed genes for 5 developmental stages of epithelial cells**

**Table S4 Antibodies used in immunofluorescence assay for marker genes**
