# Supplementary material for: A Single-cell Atlas of Developing Mouse Palates Reveals Cellular and Molecular Transitions in Periderm Cell Fate
Source: Genomics Proteomics Bioinformatics. 2025 Mar 4;23(1):qzaf013. doi: 10.1093/gpbjnl/qzaf013 (PMC12240470; doi:10.1093/gpbjnl/qzaf013)
Supplement: qzaf013_Supplementary_Data [file qzaf013_supplementary_data.zip › Table S1.docx]

**Table S1 Summary of cell clusters during mouse palatogenesis**

| **Cluster** | **Marker genes** | **Number of cells** | | | | | **Putative identity** |
| --- | --- | --- | --- | --- | --- | --- | --- |
|  |  | **E10.5 (N = 12,368)** | **E13.5 (N = 8950)** | **E15 (N = 9173)** | **E16.5 (N = 10,928)** | **Overall (N = 41,419)** |  |
| C0 | *Pax9, Barx1* | 33 (0.3%) | 1313 (14.7%) | 1145 (12.5%) | 1361 (12.5%) | 3852 (9.3%) | Developing palatal mesenchymal cells-posterior region |
| C1 | *Pax9, Barx1* | 25 (0.2%) | 872 (9.7%) | 889 (9.7%) | 1520 (13.9%) | 3306 (8.0%) | Developing palatal mesenchymal cells-posterior region |
| C2 | *Sp7, Alpl* | 43 (0.3%) | 1092 (12.2%) | 957 (10.4%) | 1172 (10.7%) | 3264 (7.9%) | Osteocyte lineage |
| C3 | *Bmp4, Msx1* | 57 (0.5%) | 588 (6.6%) | 1278 (13.9%) | 1082 (9.9%) | 3005 (7.3%) | Developing palatal mesenchymal cells-anterior region |
| C4 | *Cks2, Birc5* | 1017 (8.2%) | 704 (7.9%) | 527 (5.7%) | 562 (5.1%) | 2810 (6.8%) | Proliferating mesenchymal cells |
| C5 | *Sox2* | 1501 (12.1%) | 97 (1.1%) | 129 (1.4%) | 90 (0.8%) | 1817 (4.4%) | Epithelial progenitor cells |
| C6 | *Krt6a, Krt6b, Lypd3* | 12 (0.1%) | 628 (7.0%) | 600 (6.5%) | 285 (2.6%) | 1525 (3.7%) | *Krt6*+ cells |
| C7 | *Sox2* | 386 (3.1%) | 246 (2.7%) | 270 (2.9%) | 309 (2.8%) | 1211 (2.9%) | Epithelial progenitor cells |
| C8 | *Alas2, Hba-a1* | 25 (0.2%) | 131 (1.5%) | 209 (2.3%) | 379 (3.5%) | 744 (1.8%) | Red blood cells |
| C9 | *Crabp1* | 0 (0%) | 120 (1.3%) | 82 (0.9%) | 57 (0.5%) | 259 (0.6%) | Early-stage mesenchymal cells |
| C10 | *Col2a1, Col9a1* | 0 (0%) | 81 (0.9%) | 100 (1.1%) | 69 (0.6%) | 250 (0.6%) | Chondrocytes |
| C11 | *Elavl3* | 13 (0.1%) | 27 (0.3%) | 43 (0.5%) | 127 (1.2%) | 210 (0.5%) | Neuronal cell lineage |
| C12 | *Alas2, Hba-a1* | 2630 (21.3%) | 58 (0.6%) | 23 (0.3%) | 20 (0.2%) | 2731 (6.6%) | Red blood cells |
| C13 | *Trpm1, Six6, Aldh1a3* | 387 (3.1%) | 447 (5.0%) | 628 (6.8%) | 971 (8.9%) | 2433 (5.9%) | Eyes-related epithelial cells |
| C14 | *-* | 1834 (14.8%) | 72 (0.8%) | 29 (0.3%) | 34 (0.3%) | 1969 (4.8%) | Ambiguous cell type |
| C15 | *Egfl7, Cdh5* | 1031 (8.3%) | 56 (0.6%) | 41 (0.4%) | 91 (0.8%) | 1219 (2.9%) | Endothelial cells |
| C16 | *Alas2, Hba-a1* | 420 (3.4%) | 113 (1.3%) | 75 (0.8%) | 134 (1.2%) | 742 (1.8%) | Red blood cells |
| C17 | *Plp1, Mpz* | 56 (0.5%) | 91 (1.0%) | 132 (1.4%) | 202 (1.8%) | 481 (1.2%) | Schwann cells |
| C18 | *Myod1, Myog* | 11 (0.1%) | 120 (1.3%) | 37 (0.4%) | 20 (0.2%) | 188 (0.5%) | Myogenic precursor cells |
| C19 | *Trpm1, Six6, Aldh1a3* | 0 (0%) | 0 (0%) | 1 (0.0%) | 83 (0.8%) | 84 (0.2%) | Eyes-related epithelial cells |
| C20 | *Alas2, Hba-a1* | 56 (0.5%) | 470 (5.3%) | 661 (7.2%) | 688 (6.3%) | 1875 (4.5%) | Red blood cells |
| C21 | *Cd74, Rac2* | 36 (0.3%) | 495 (5.5%) | 392 (4.3%) | 350 (3.2%) | 1273 (3.1%) | Hematopoietic progenitor cells |
| C22 | *Lypd2, Cbr2* | 659 (5.3%) | 109 (1.2%) | 124 (1.4%) | 90 (0.8%) | 982 (2.4%) | Nasal epithelial cells |
| C23 | *Elavl3* | 639 (5.2%) | 19 (0.2%) | 27 (0.3%) | 23 (0.2%) | 708 (1.7%) | Neuronal cell lineage |
| C24 | *Sp7, Alpl* | 863 (7.0%) | 189 (2.1%) | 116 (1.3%) | 213 (1.9%) | 1381 (3.3%) | Osteocyte lineage |
| C25 | *Bmp4, Msx1* | 188 (1.5%) | 161 (1.8%) | 62 (0.7%) | 48 (0.4%) | 459 (1.1%) | Developing palatal mesenchymal cells-anterior region |
| C26 | *Myl9* | 304 (2.5%) | 282 (3.2%) | 181 (2.0%) | 300 (2.7%) | 1067 (2.6%) | Smooth muscle cells |
| C27 | *Krt14* | 26 (0.2%) | 281 (3.1%) | 209 (2.3%) | 433 (4.0%) | 949 (2.3%) | Epithelial basal cells |
| C28 | *Ambn, Amelx* | 116 (0.9%) | 88 (1.0%) | 206 (2.2%) | 215 (2.0%) | 625 (1.5%) | Dental cells |

*Note*: The percentages in parentheses indicate the proportion of each cluster at the corresponding time point. E, embryonic day.
