# Supplementary material for: A Single-cell Atlas of Developing Mouse Palates Reveals Cellular and Molecular Transitions in Periderm Cell Fate
Source: Genomics Proteomics Bioinformatics. 2025 Mar 4;23(1):qzaf013. doi: 10.1093/gpbjnl/qzaf013 (PMC12240470; doi:10.1093/gpbjnl/qzaf013)
Supplement: qzaf013_Supplementary_Data [file qzaf013_supplementary_data.zip › Table S4.docx]

**Table S4 Antibodies used in immunofluorescence assay for marker genes**

| **Marker** | **Clusters** | **Cell type** | **Company** | **Catalog No.** | **Dilution** | **Ref.** |
| --- | --- | --- | --- | --- | --- | --- |
| COL3A1 | C0–C4, C9, C10, C14, C18, C24, C25, C26 | Mesenchymal cells | Proteintech | 22734-1-AP | 1:100 | [15] |
| TRP63 | C5–C7, C13, C19, C22, C27–28 | Epithelial cells | Proteintech | 12143-1-AP | 1:800 | [16] |
| KRT6A | C6 | *Krt6*+ cells | Proteintech | 10590-1-AP | 1:200 | [37] |
| KRT10 | C6.5.0 | Keratinized periderm cells I | Proteintech | 18343-1-AP | 1:100 | [46] |
| KLF4 | C6.5.1 | Keratinized periderm cells II | Proteintech | 11880-1-AP | 1:100 | [16] |
| IGFBP3 | C6.5.2 | Medial edge periderm cells | Proteintech | 10189-2-AP | 1:100 | [55] |
| CLDN3 | C6.5.3 | Primitive periderm cells | Proteintech | 16456-1-AP | 1:100 | [14] |
| ARHGAP29 | C6.5.3 | Primitive periderm cells | Proteintech | 12583-1-AP | 1:100 | [44] |
| STMN1 | C6.5.2 | Medial edge periderm cells | CST | 3352 | 1:100 | [58] |
| ANXA6 | C6.5.2 | Medial edge periderm cells | Proteintech | 12542-1-AP | 1:100 | [59] |
| TAGLN | C6.5.2 | Medial edge periderm cells | CST | 40471 | 1:100 | [60] |
