# Supplementary figures and images for: A Single-cell Atlas of Developing Mouse Palates Reveals Cellular and Molecular Transitions in Periderm Cell Fate
Source: Genomics Proteomics Bioinformatics. 2025 Mar 4;23(1):qzaf013. doi: 10.1093/gpbjnl/qzaf013 (PMC12240470; doi:10.1093/gpbjnl/qzaf013)

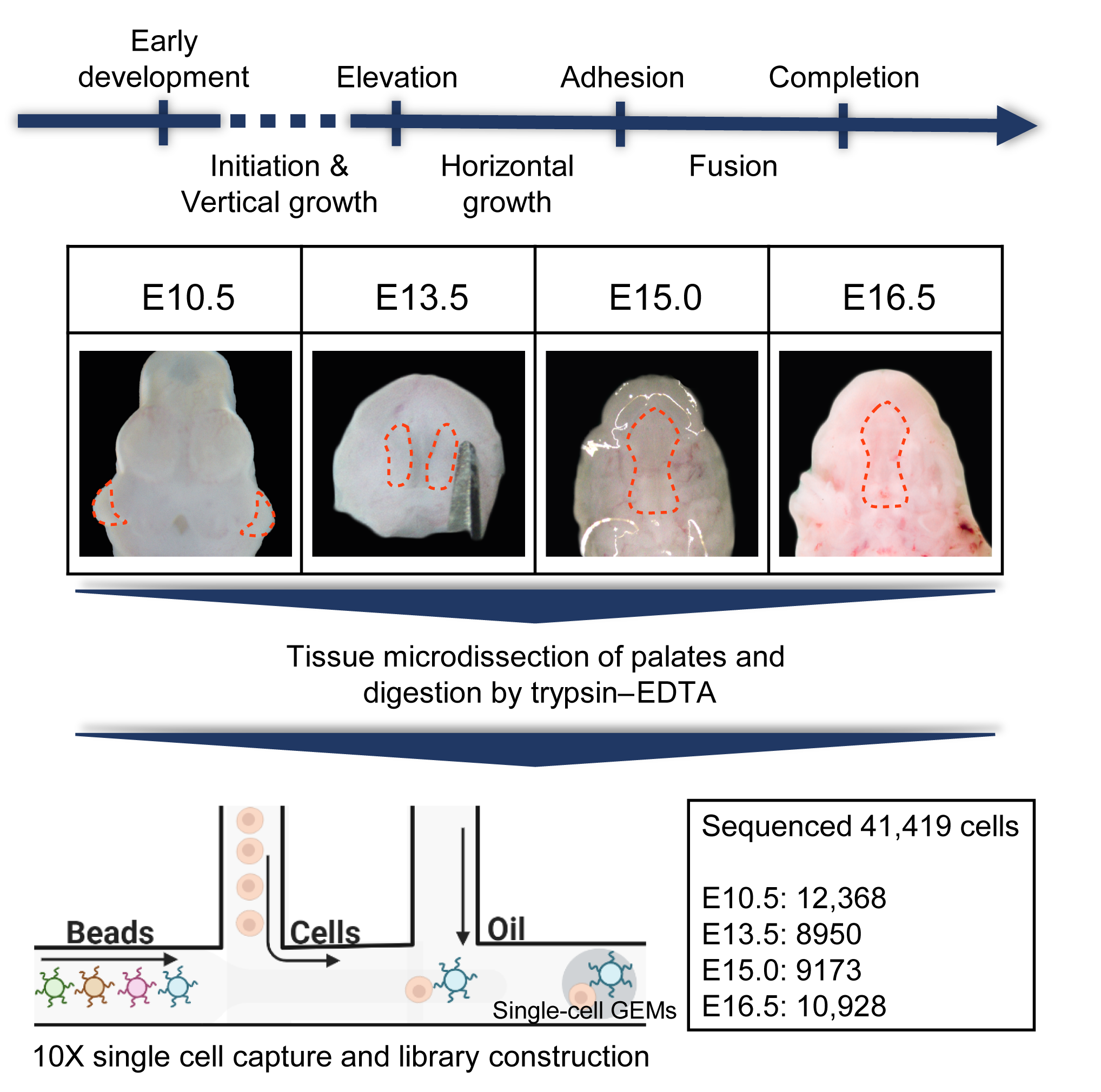

Supplement: qzaf013_Supplementary_Data [file qzaf013_supplementary_data.zip › Figure S1.tif]

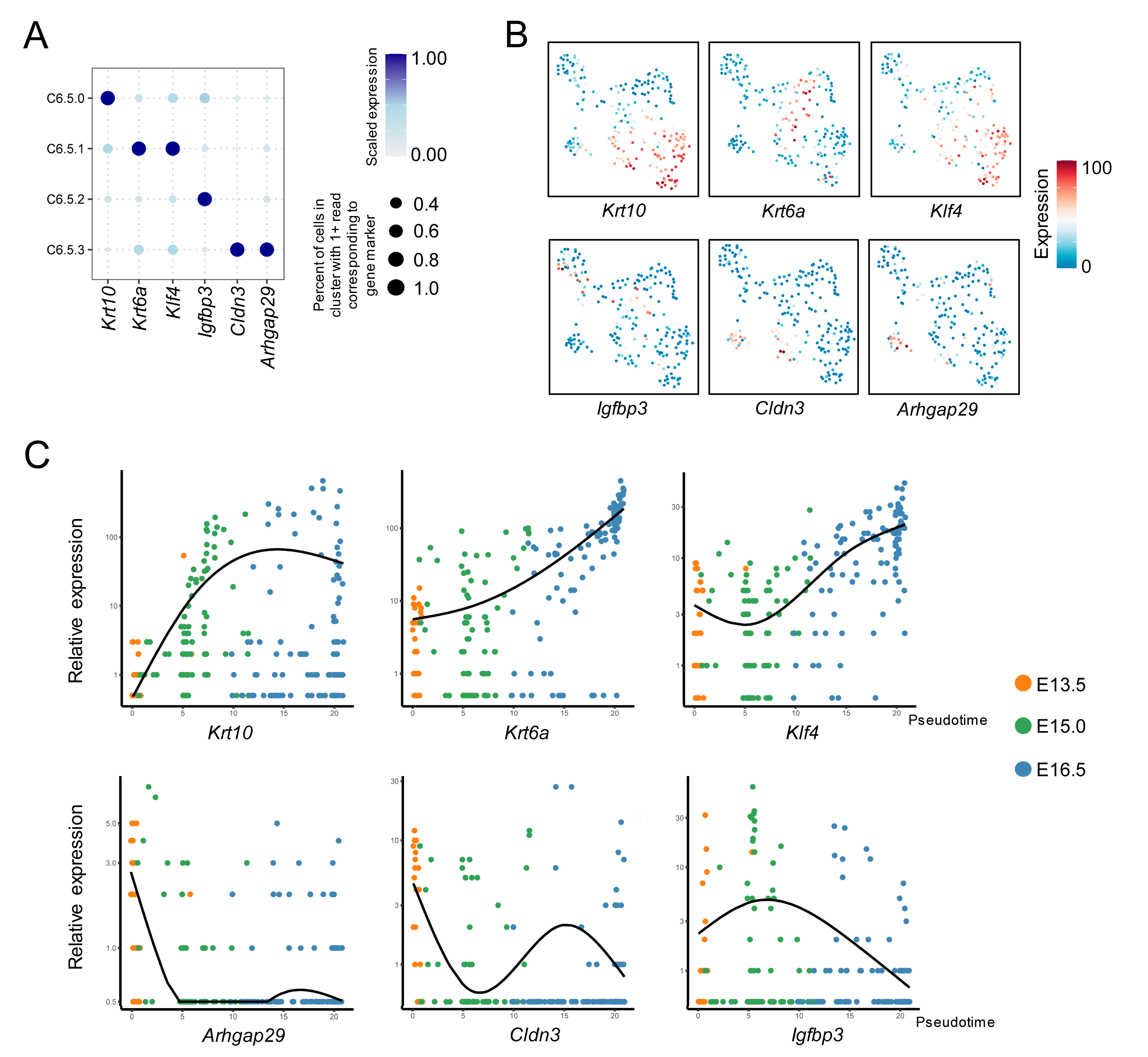

Supplement: qzaf013_Supplementary_Data [file qzaf013_supplementary_data.zip › Figure S10.tif]

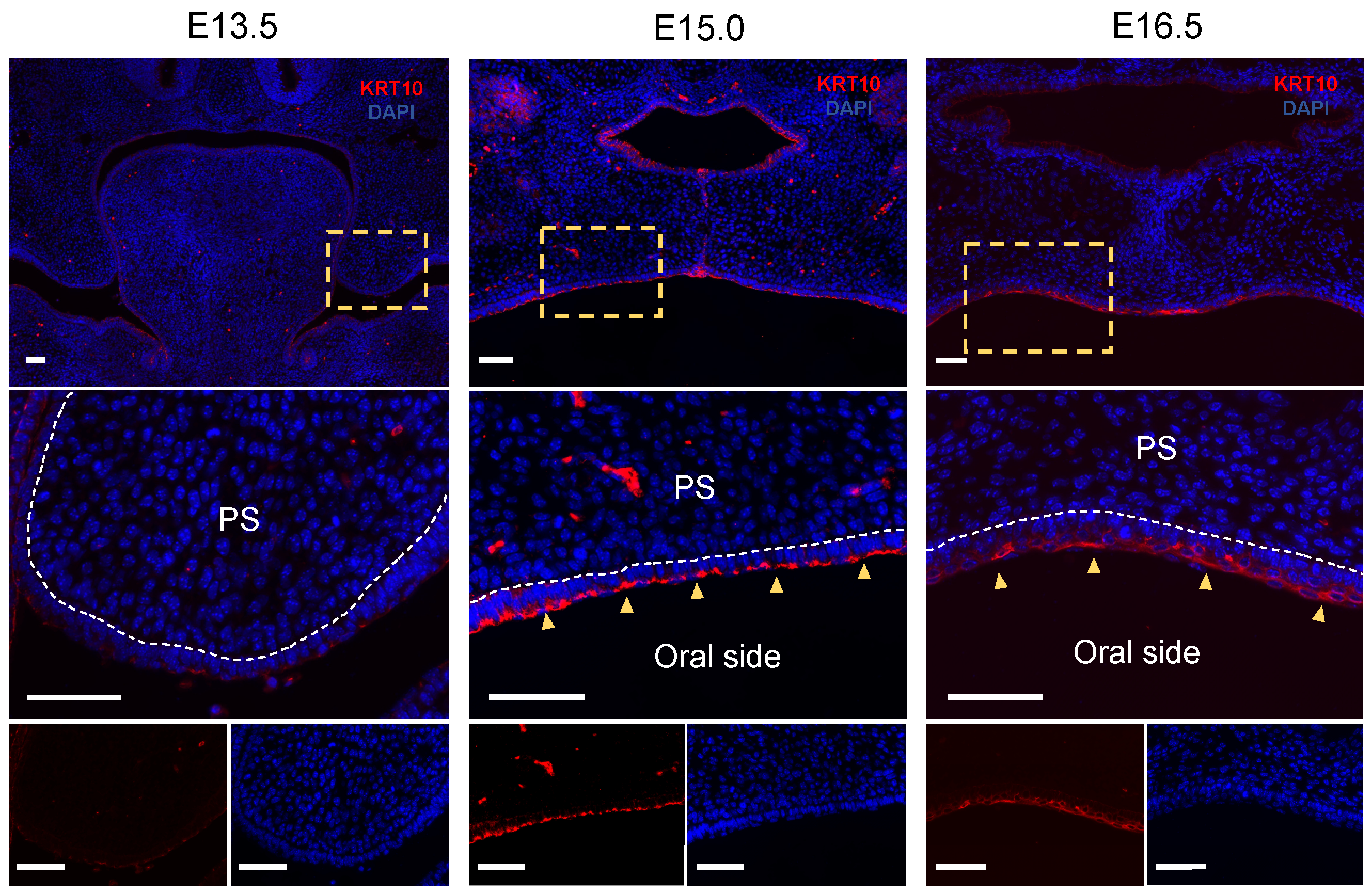

Supplement: qzaf013_Supplementary_Data [file qzaf013_supplementary_data.zip › Figure S11.tif]

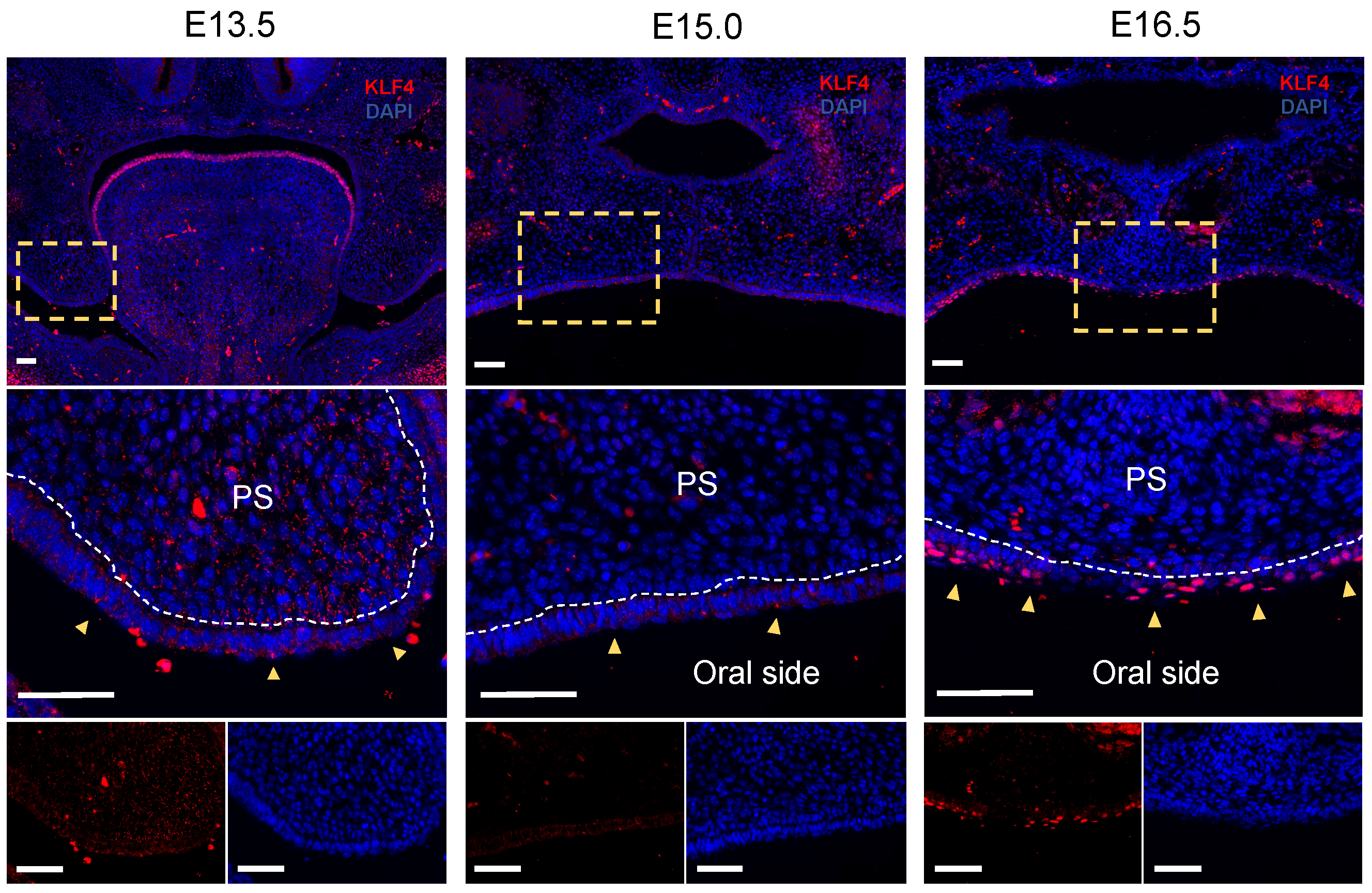

Supplement: qzaf013_Supplementary_Data [file qzaf013_supplementary_data.zip › Figure S12.tif]

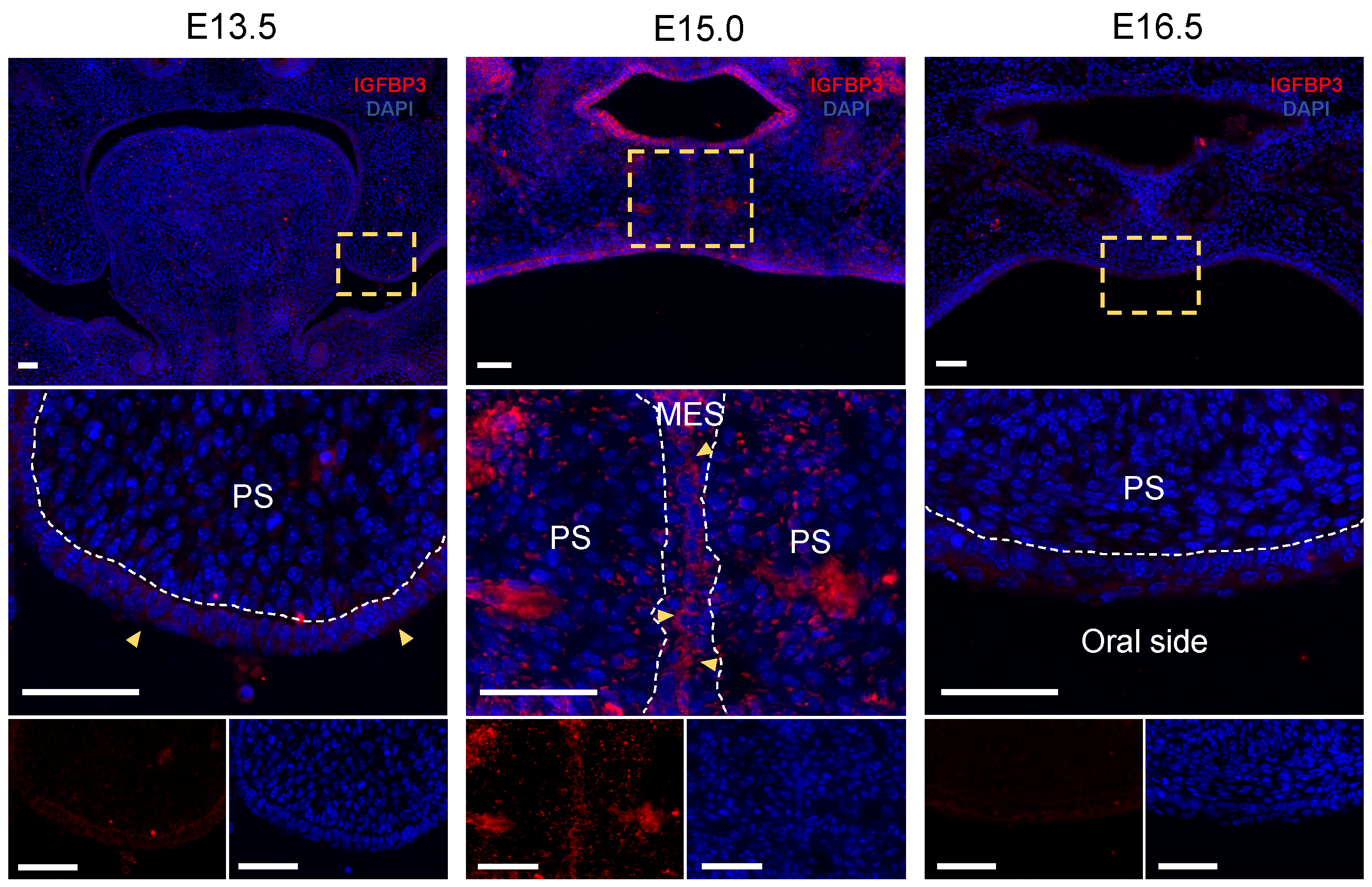

Supplement: qzaf013_Supplementary_Data [file qzaf013_supplementary_data.zip › Figure S13.tif]

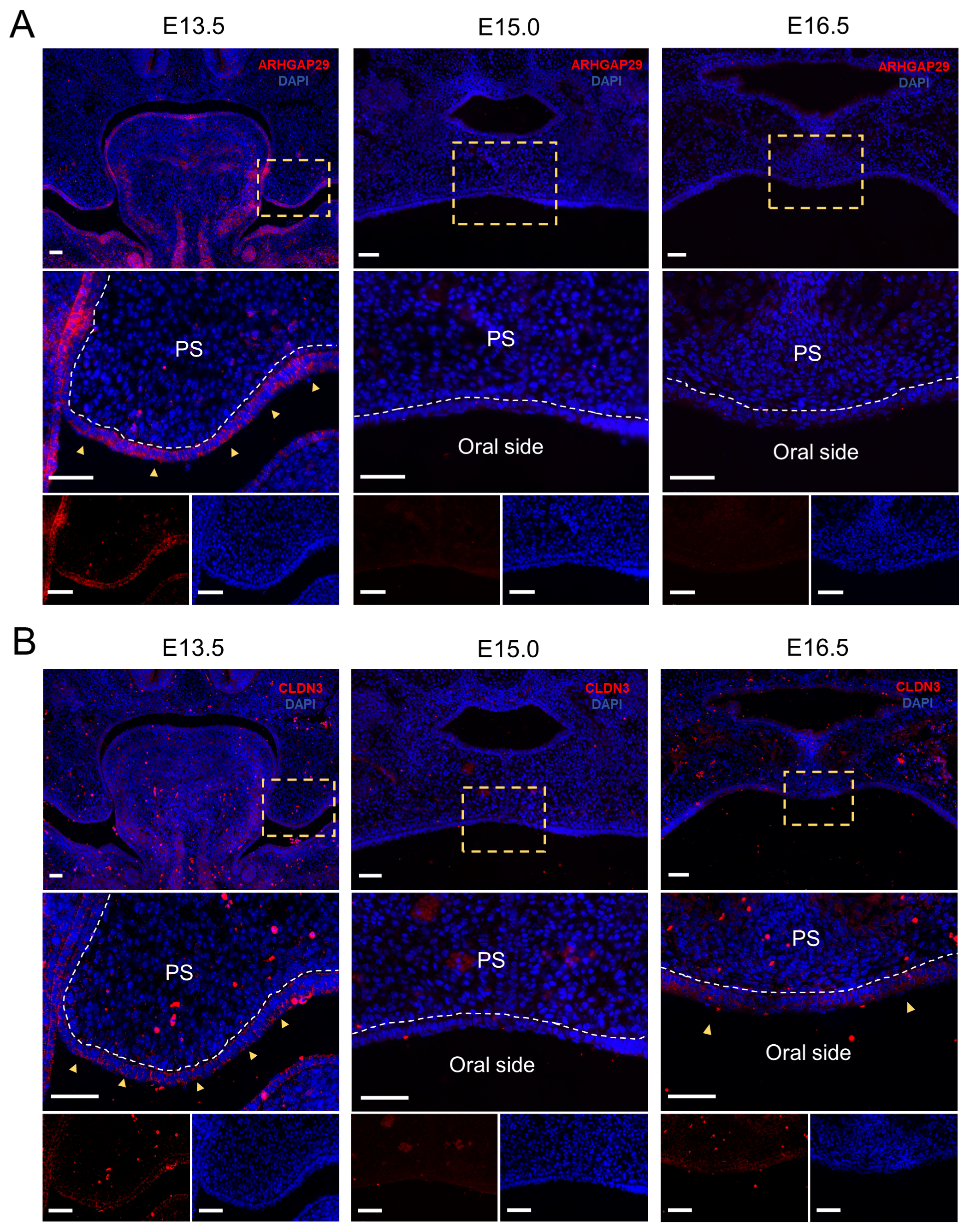

Supplement: qzaf013_Supplementary_Data [file qzaf013_supplementary_data.zip › Figure S14.tif]

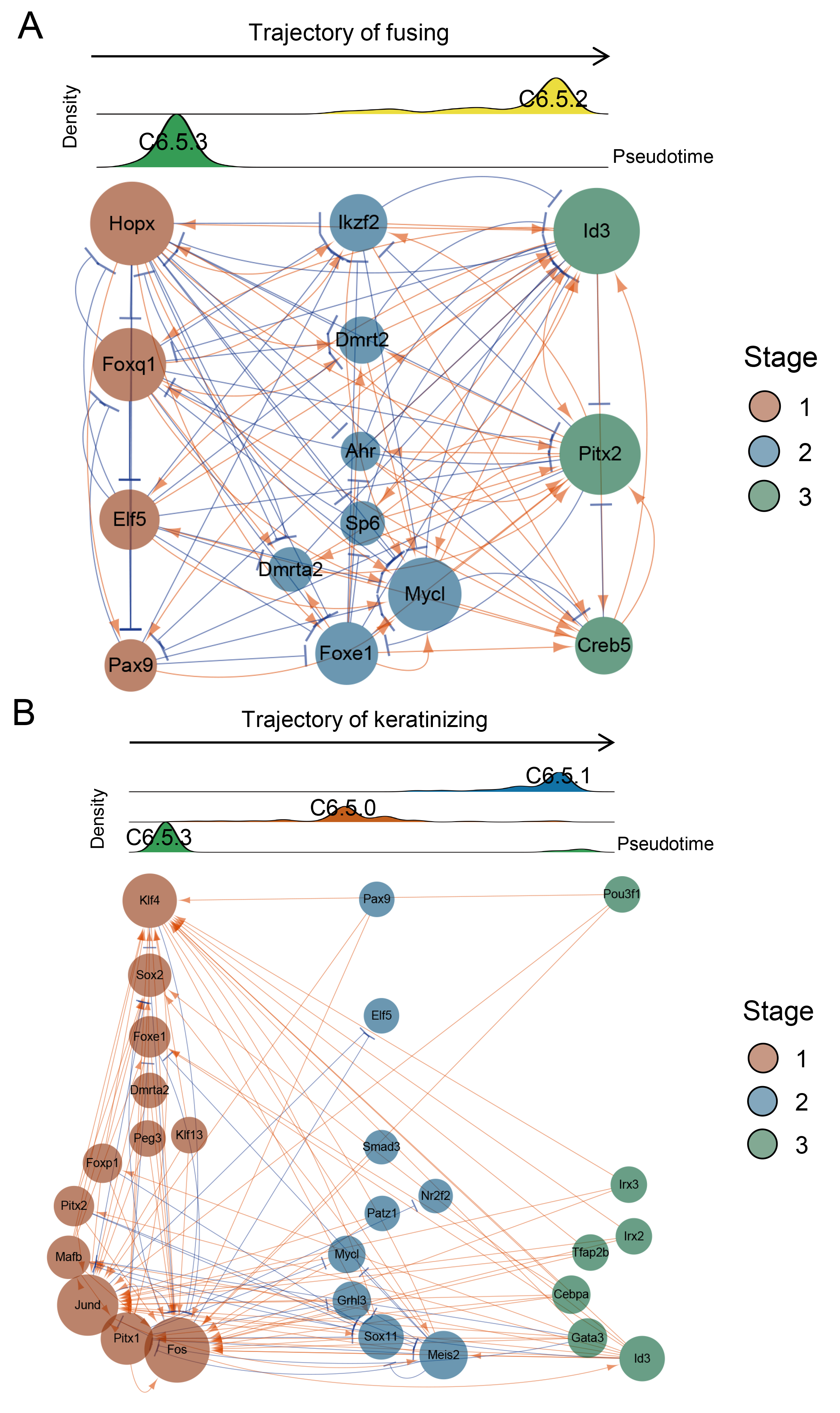

Supplement: qzaf013_Supplementary_Data [file qzaf013_supplementary_data.zip › Figure S15.tif]

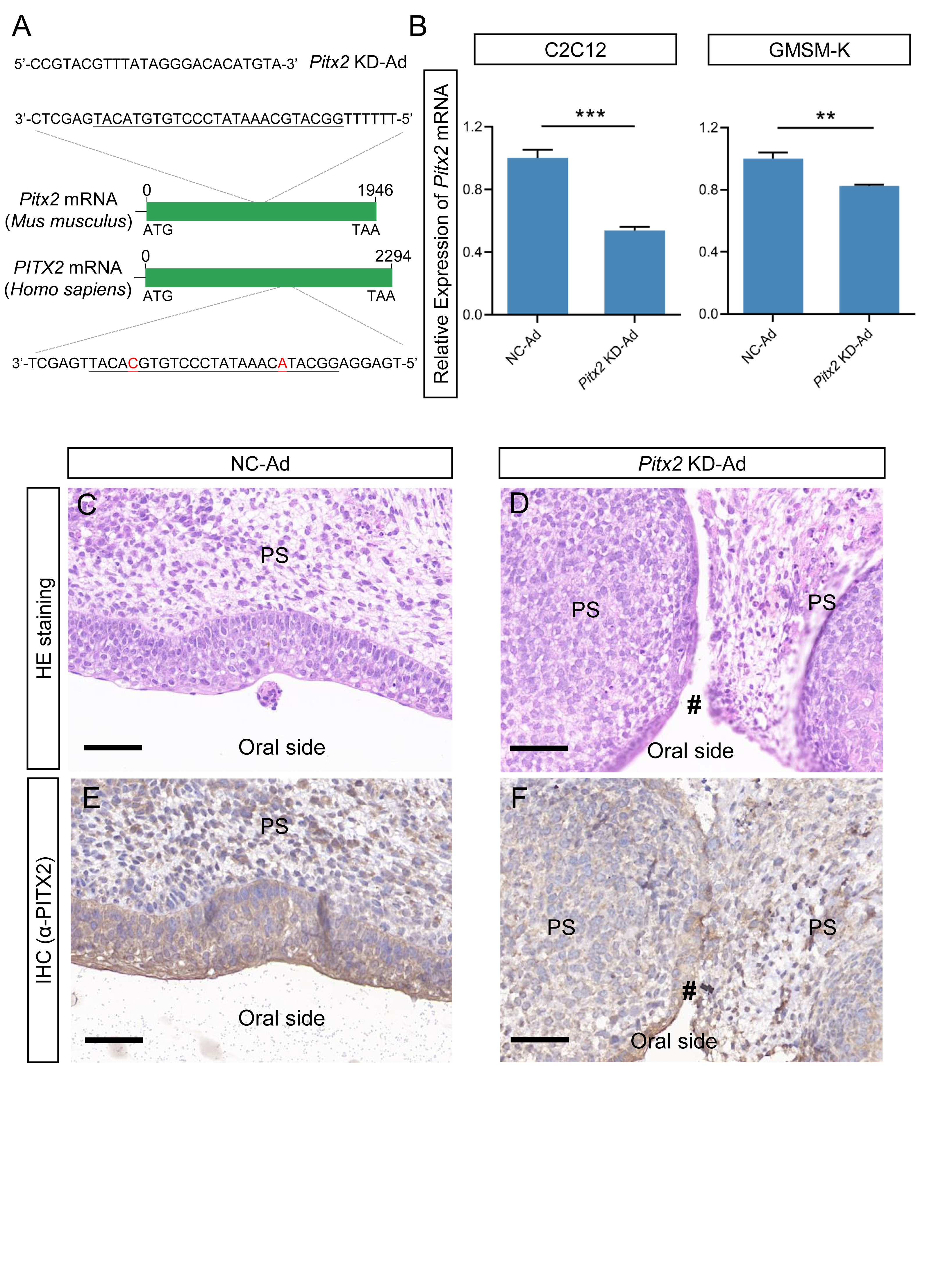

Supplement: qzaf013_Supplementary_Data [file qzaf013_supplementary_data.zip › Figure S16.tif]

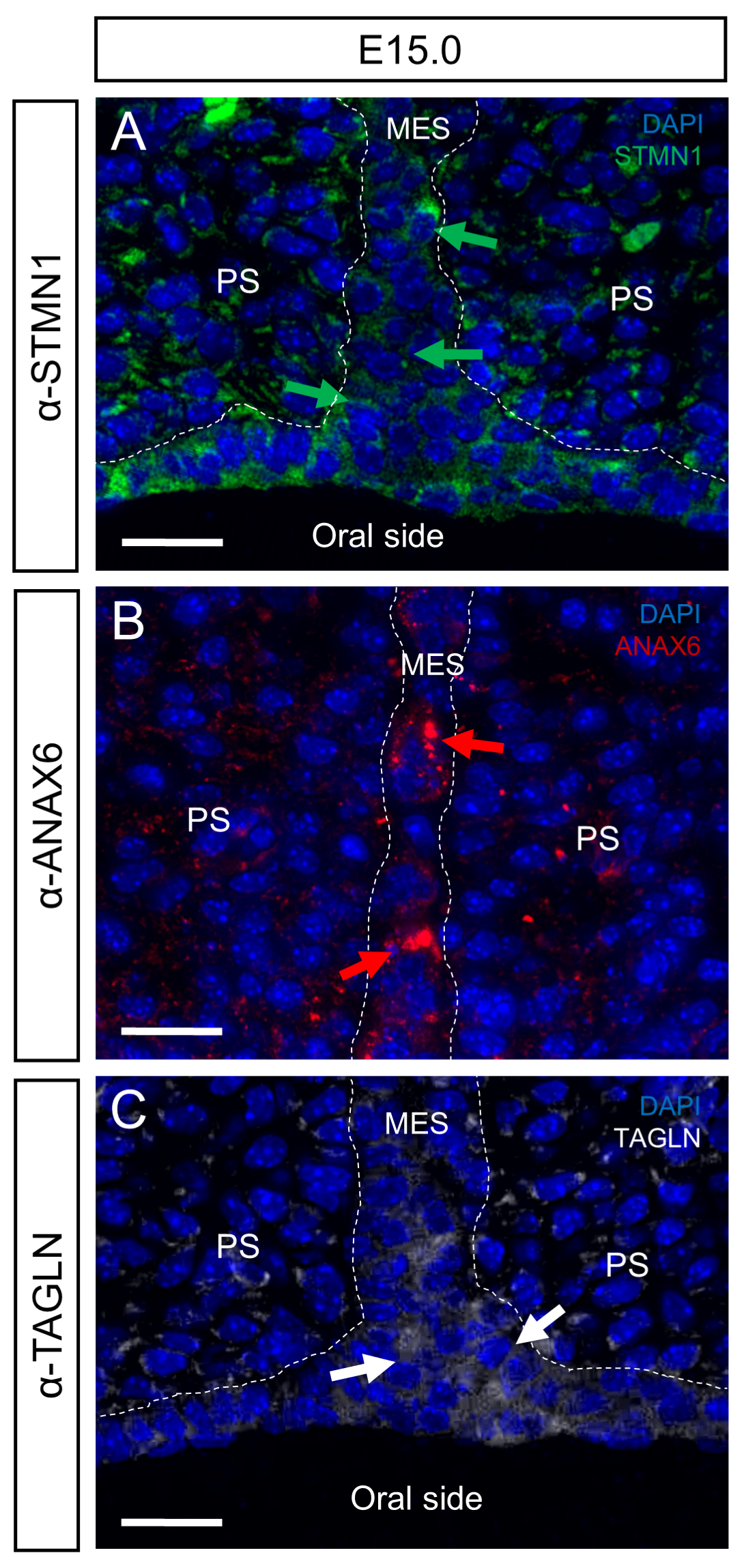

Supplement: qzaf013_Supplementary_Data [file qzaf013_supplementary_data.zip › Figure S17.tif]

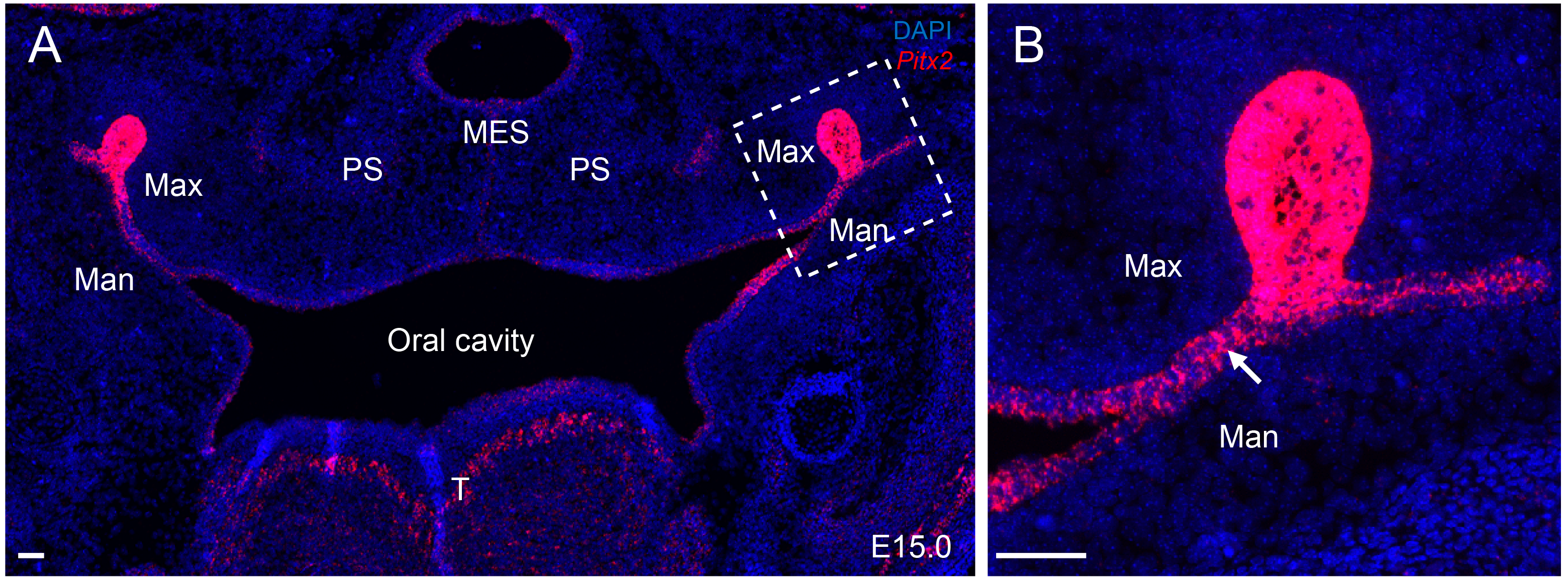

Supplement: qzaf013_Supplementary_Data [file qzaf013_supplementary_data.zip › Figure S18.tif]

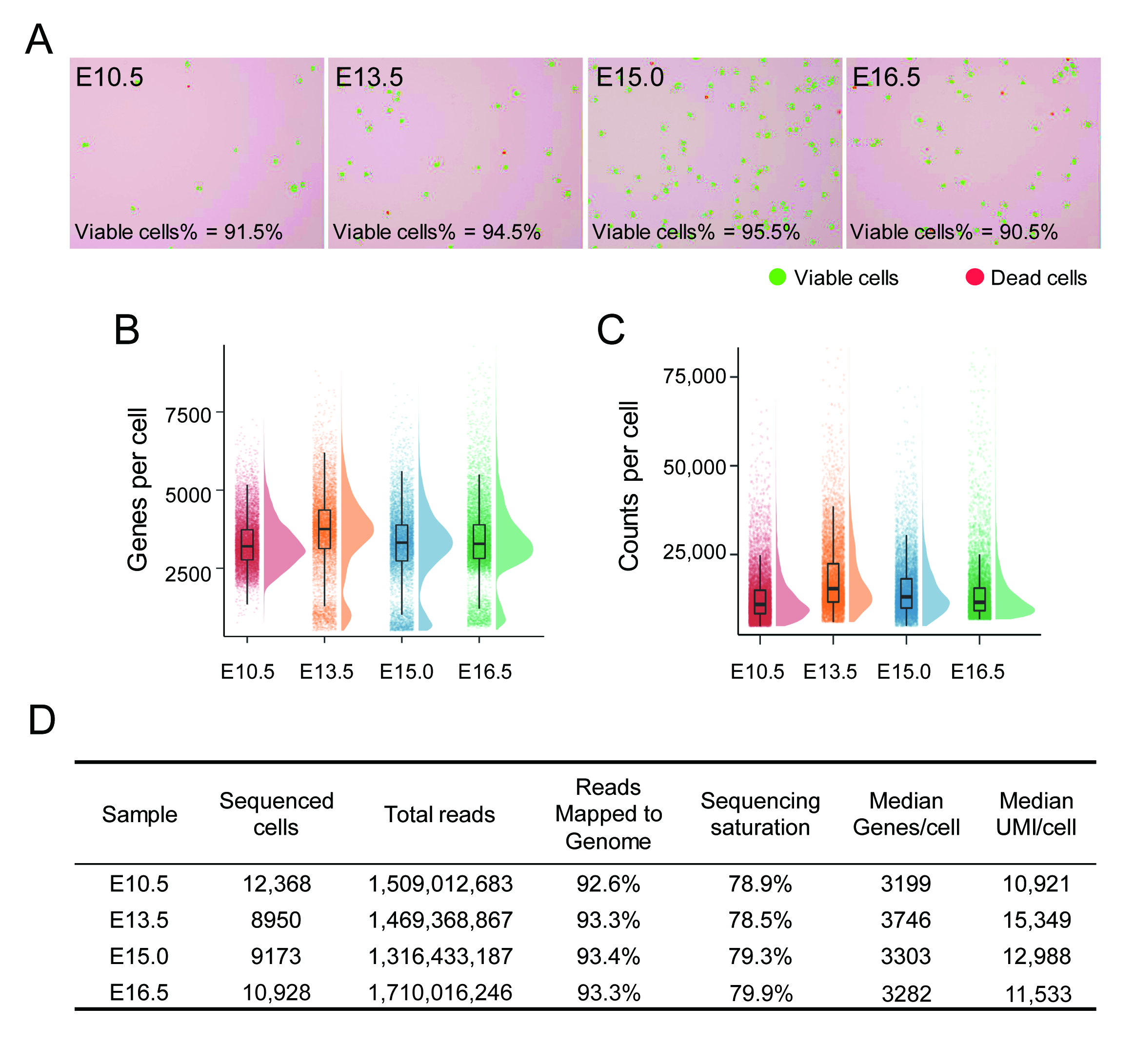

Supplement: qzaf013_Supplementary_Data [file qzaf013_supplementary_data.zip › Figure S2.tif]

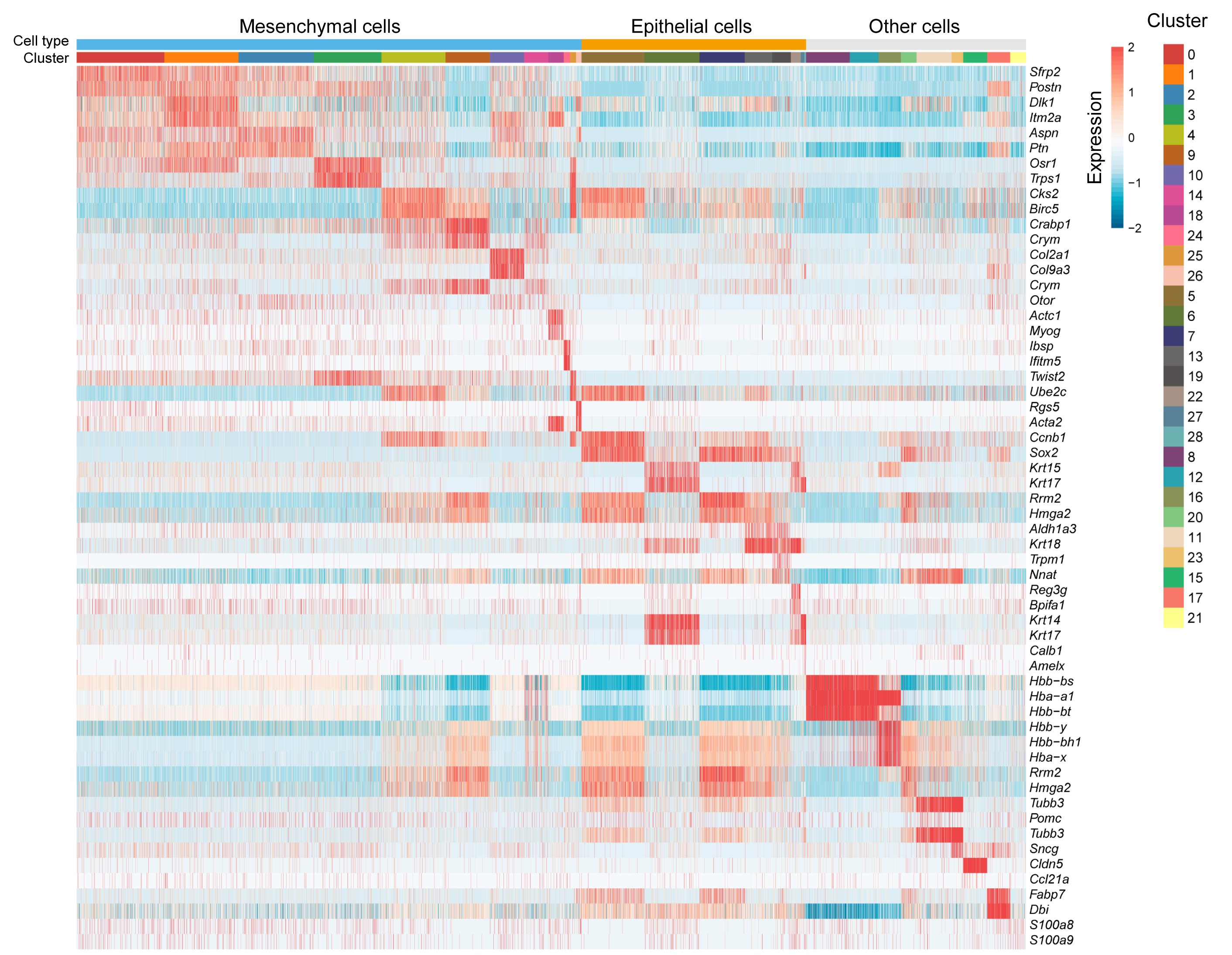

Supplement: qzaf013_Supplementary_Data [file qzaf013_supplementary_data.zip › Figure S3.tif]

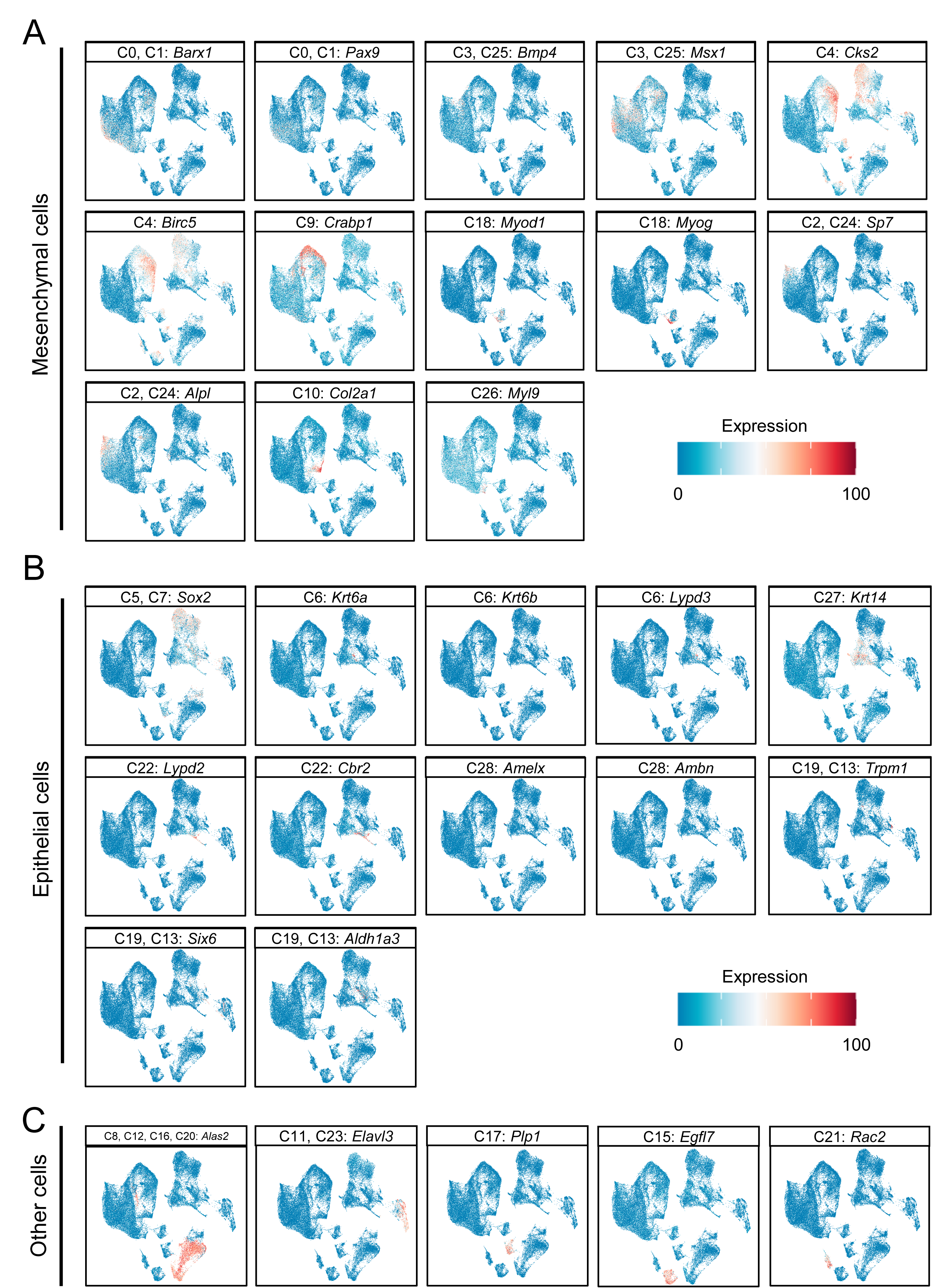

Supplement: qzaf013_Supplementary_Data [file qzaf013_supplementary_data.zip › Figure S4.tif]

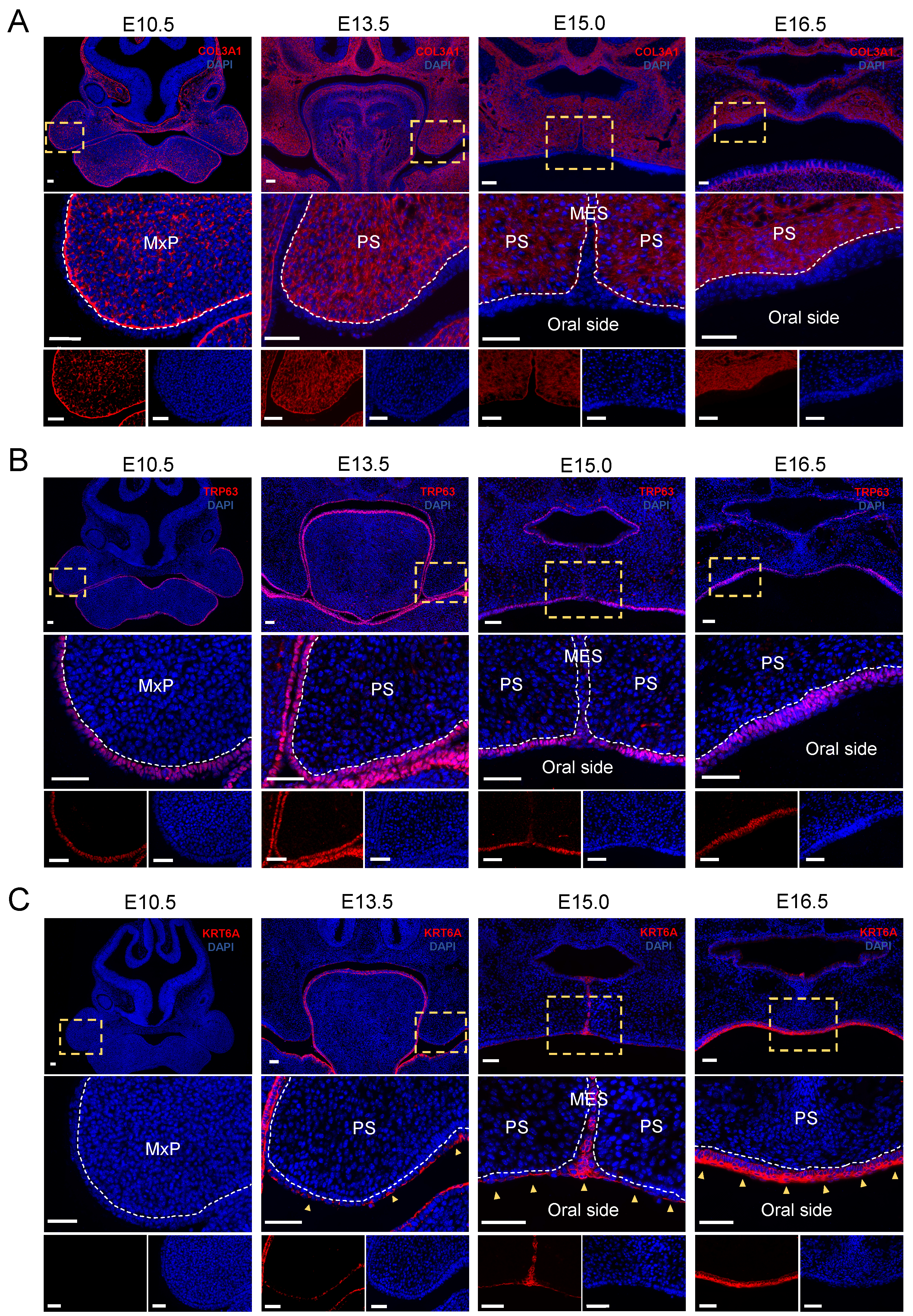

Supplement: qzaf013_Supplementary_Data [file qzaf013_supplementary_data.zip › Figure S5.tif]

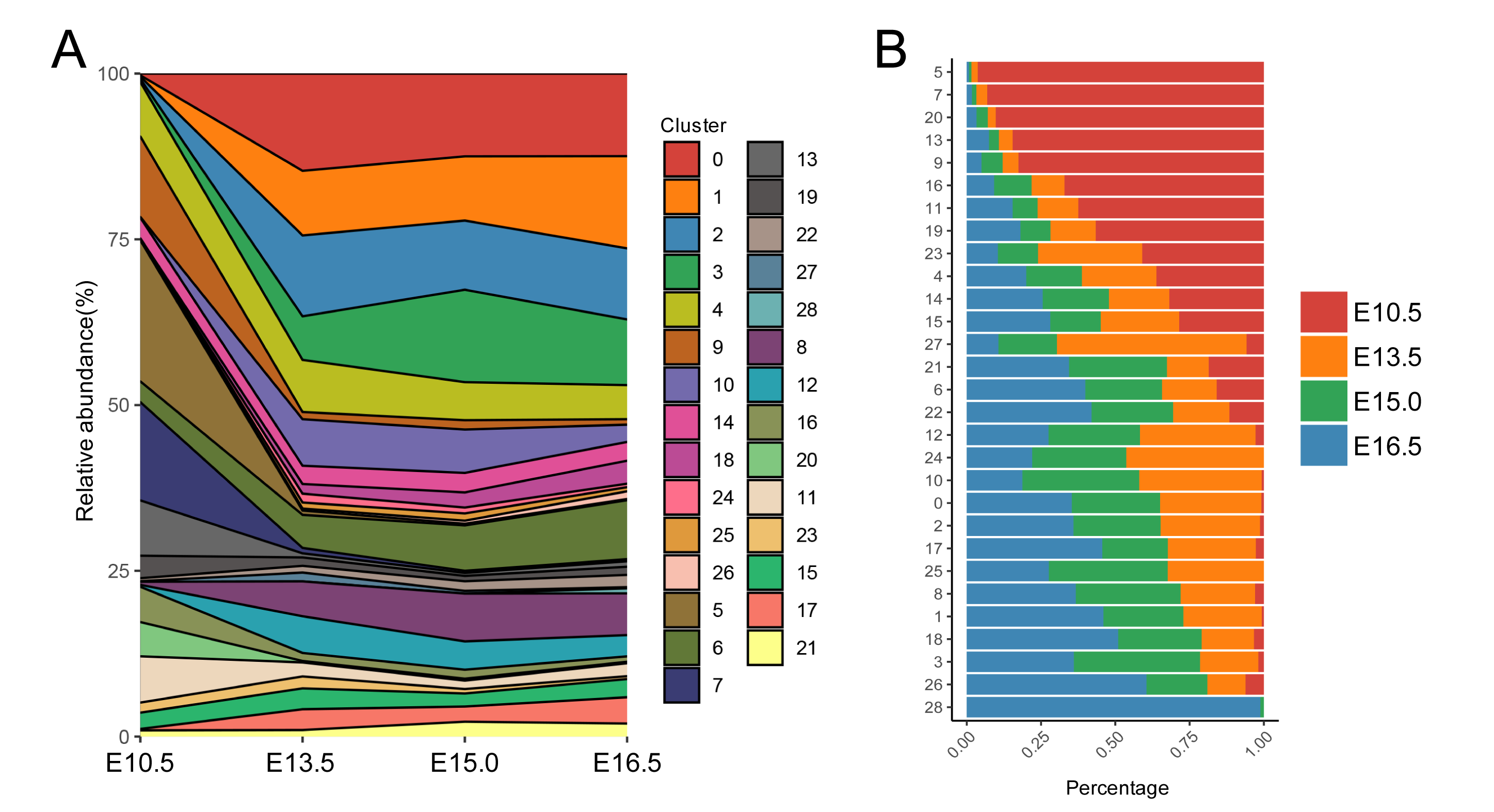

Supplement: qzaf013_Supplementary_Data [file qzaf013_supplementary_data.zip › Figure S6.tif]

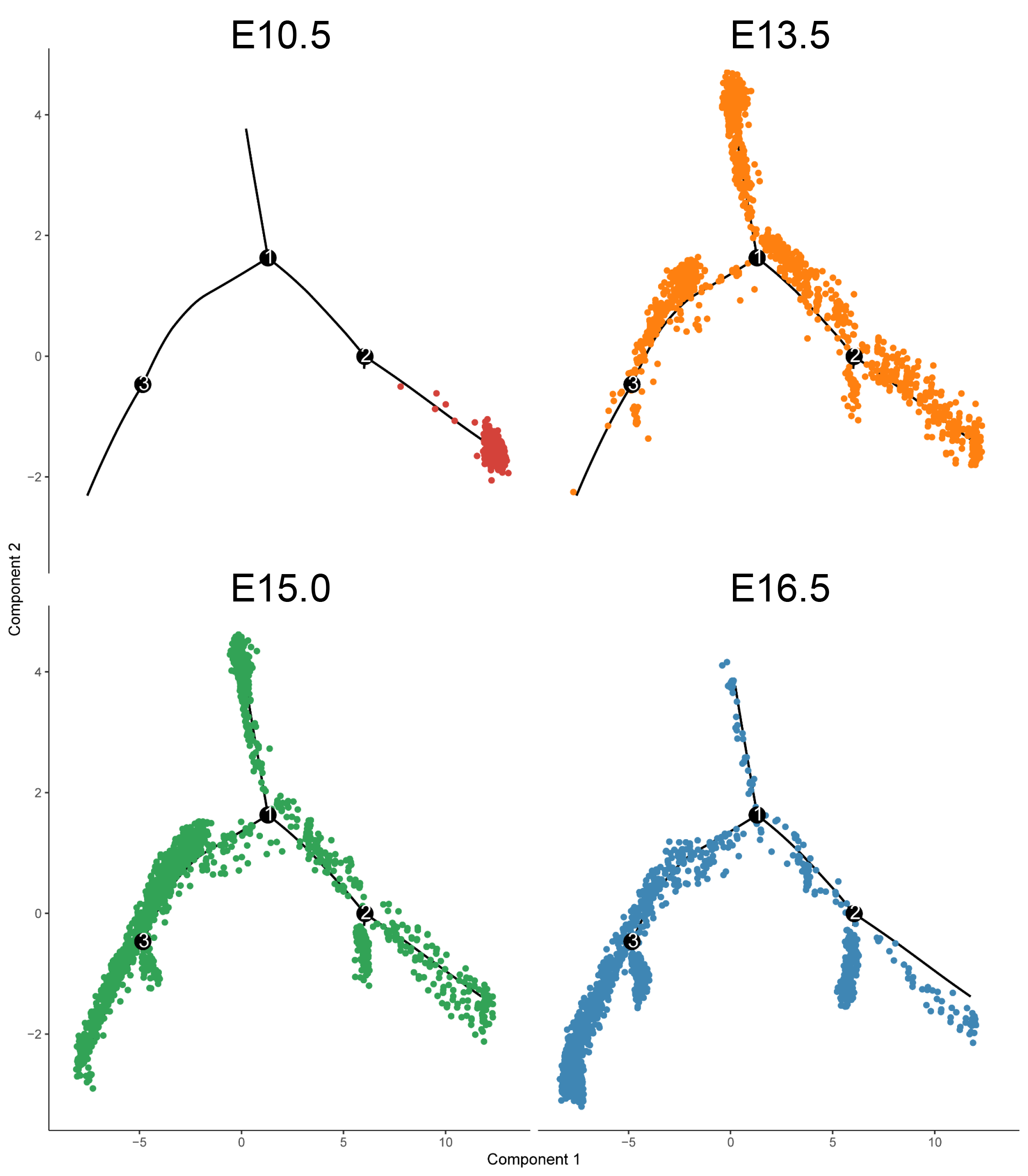

Supplement: qzaf013_Supplementary_Data [file qzaf013_supplementary_data.zip › Figure S7.tif]

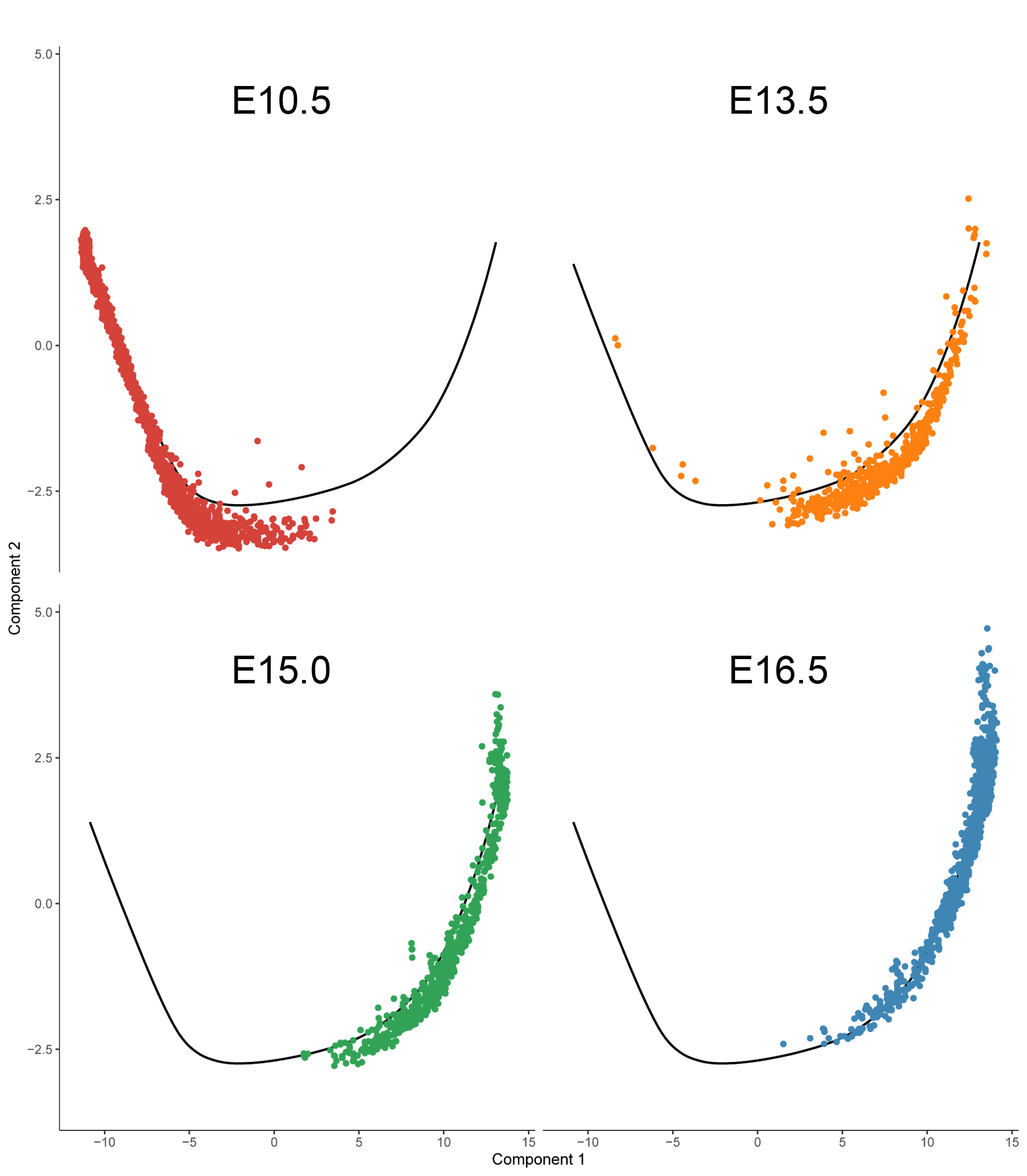

Supplement: qzaf013_Supplementary_Data [file qzaf013_supplementary_data.zip › Figure S8.tif]

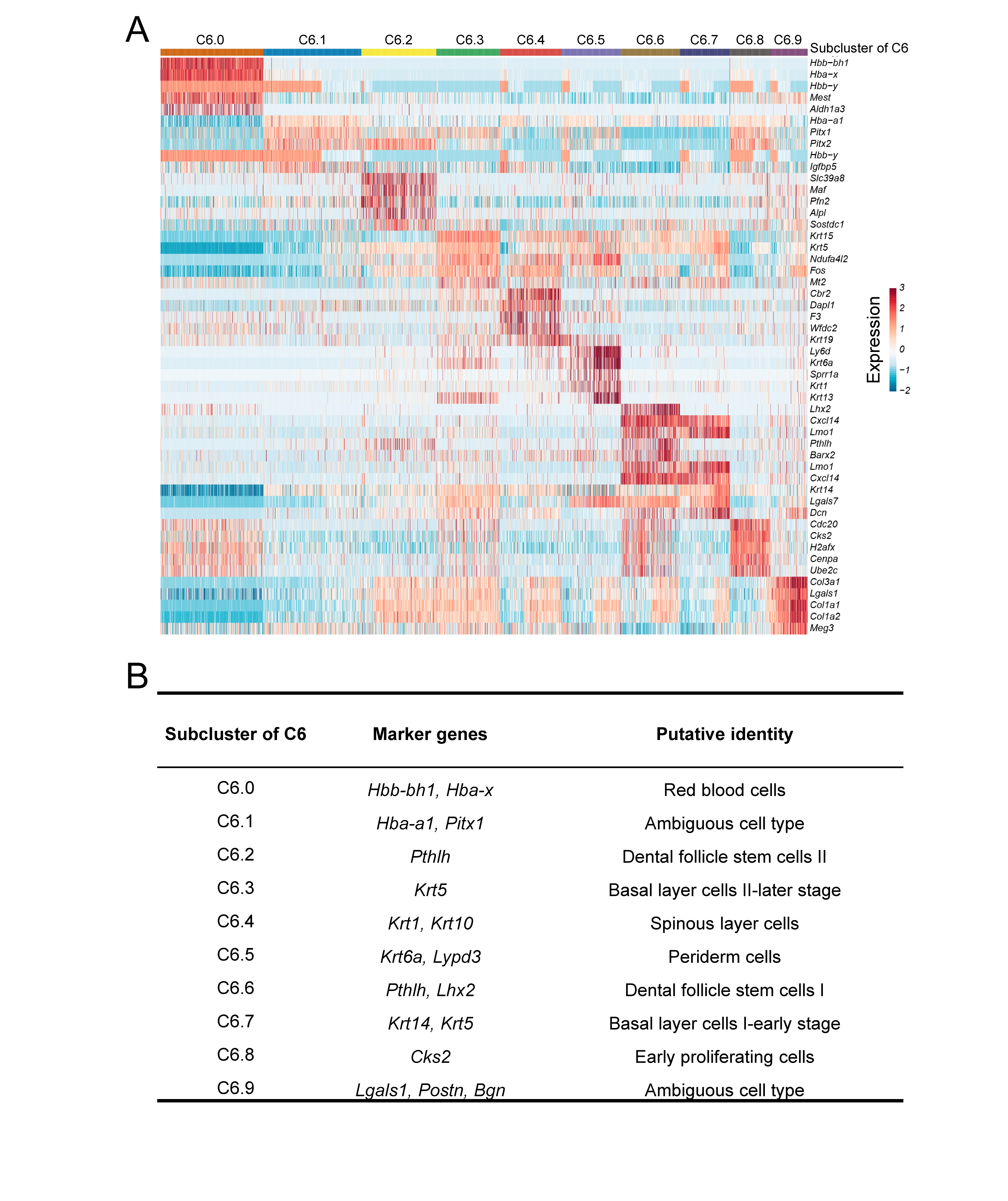

Supplement: qzaf013_Supplementary_Data [file qzaf013_supplementary_data.zip › Figure S9.tif]
